# Single odor identification in subjective cognitive decline, mild cognitive impairment and Alzheimer’s dementia

**DOI:** 10.1007/s00508-026-02721-z

**Published:** 2026-03-09

**Authors:** Abdallah Tasnim, Johann Lehrner

**Affiliations:** Department of Neurology, Medical University of Vienna, Währinger Gürtel 18–20, 1090 Vienna, Austria

**Keywords:** Early diagnosis, Item-specific analysis, Smell disorder, Elderly

## Abstract

**Background:**

Olfactory dysfunction is an early biomarker of Alzheimer’s dementia (AD) and cognitive impairments. This study examines odor-specific olfactory deficits across cognitive impairment stages using the Sniffin’ Sticks Odor Identification Test (SS-OIT).

**Objective:**

To assess the discriminative validity of specific odors in differentiating subjective cognitive decline (SCD), non-amnestic mild cognitive impairment (naMCI), amnestic mild cognitive impairment (aMCI), and AD from healthy controls (HC). Additionally, the influences of age, gender, verbal intelligence and depression on olfactory performance were analyzed. Participants were also grouped into an AD versus non-AD category (comprising SCD, naMCI, and aMCI) for logistic regression analyses.

**Methods:**

A retrospective analysis at the Department of Neurology of the Medical University of Vienna included 737 participants aged 50 years and older. Olfactory identification was assessed using the 16-item Sniffin’ Sticks odor identification test. The discriminative power of specific odors was evaluated for distinguishing AD from non-AD groups. Bonferroni corrections were applied to adjust for multiple comparisons, which increases the stringency of statistical significance across the 16 odors tested.

**Results:**

Significant differences in odor identification were observed across diagnostic groups. The AD patients exhibited the most pronounced deficits, particularly in recognizing clove, rose and aniseed. Age negatively correlated with olfactory performance, while higher verbal intelligence was a protective factor. Key odors differentiated AD from non-AD groups, highlighting the diagnostic potential of olfactory testing.

**Conclusion:**

Odor-specific olfactory deficits serve as early indicators of cognitive decline. The Sniffin’ Sticks test, particularly key odors, may aid early detection and differentiation of cognitive impairments. Accounting for covariates enhances the diagnostic accuracy. Future research should aim to refine olfactory testing protocols and assess their clinical utility in broader populations.

## Introduction

Alzheimer’s disease (AD) is the leading cause of dementia and cognitive decline in older adults. It progresses gradually, with early pathological changes, including amyloid-beta (Aβ) plaques, neurofibrillary tangles and neuroinflammation, leading to neuronal damage and synaptic dysfunction [1]. Given the irreversible nature of AD, early detection is essential for timely intervention. Subjective cognitive decline (SCD) is now recognized as a potential precursor stage, where individuals report memory issues despite normal cognitive test results. While once considered a part of normal aging, SCD is now linked to AD biomarkers, increasing its significance as an early indicator of cognitive impairment [2]. Mild cognitive impairment (MCI), an intermediate stage between normal aging and dementia, is characterized by objective cognitive deficits that do not yet impair daily function. A distinction is typically made between amnestic (aMCI), which primarily affects memory and non-amnestic MCI (naMCI), which affects other cognitive domains. This distinction is relevant because aMCI carries a higher risk of progression to AD. With an annual conversion rate to AD of approximately 15%, MCI represents a critical stage for early diagnosis and intervention [3]. Recent research has highlighted olfactory dysfunction as an early, noninvasive biomarker for AD. Studies indicate that between 3.8% and 5.8% of the general population experience olfactory impairment, rising to 13.9% in those over 65 years old [4]. Importantly, 85–90% of AD patients exhibit significant olfactory deficits, suggesting early pathological involvement of olfactory structures [5].

Neuropathological studies confirm early tau protein accumulation in the olfactory bulb and related brain regions in MCI, reinforcing the link between olfactory impairment and disease progression [6]. These olfactory-related brain regions such as the piriform cortex, amygdala and entorhinal cortex are directly connected to memory-related areas, including the hippocampus, which is among the first regions affected by AD pathology [8]. This neuroanatomical overlap may explain why olfactory dysfunction often precedes measurable cognitive decline. Olfactory dysfunction in AD primarily affects odor detection, discrimination and identification [7]. In this study, odor identification is the primary focus as it is more robustly linked to cognitive performance than mere odor detection; however, the mechanism behind odor-specific vulnerabilities remains poorly understood. Olfactory testing is a promising early diagnostic tool. Impaired odor identification has been associated with AD progression, and when combined with cognitive assessments like the Mini-Mental State Examination (MMSE), diagnostic accuracy improves. Despite strong evidence, olfactory testing remains underutilized due to methodological inconsistencies and the absence of standardized protocols; however, the Sniffin’ Sticks Odor Identification Test (SS-OIT, Burghart Messtechnik GmbH, Holm, Germany) has demonstrated reliability in detecting early olfactory decline, making it a viable screening method [10]. Studies indicate that specific odor identification deficits may serve as early markers of cognitive decline. Research highlights that peppermint, cinnamon, clove, banana, coffee and roses are among the most affected odors in AD and MCI patients [11]. Notably, impaired recognition of peppermint has been linked to an increased risk of dementia, while cinnamon and clove have been identified as sensitive markers for AD [6]. Certain odors may be more robust indicators because of their cultural familiarity, semantic associations and distinctiveness, which are less susceptible to decline until later disease stages. Furthermore, a study on the q‑Sticks test, a brief olfactory screening tool, identified clove, coffee and rose as reliable odors due to their high familiarity and stable identification rates across ages. The test effectively distinguished anosmic, hyposmic and normosmic individuals, with 96% specificity for detecting anosmia. While not a replacement for full olfactory assessments, its ease of use and reusability make it a practical tool for identifying olfactory impairments linked to neurodegenerative diseases [12].

Most previous studies have focused on total olfactory scores but fewer have examined the discriminative ability of individual odorants across cognitive stages. This study seeks to address this gap by analyzing odor-specific deficits. Additionally, it incorporates verbal intelligence and depressive symptoms, both known to influence olfactory performance, as covariates in the analysis. Verbal intelligence may reflect cognitive reserve and support better odor identification, while depression has been linked to impaired olfactory processing independent of AD pathology.

This study aims to investigate odor-specific olfactory deficits across different stages of cognitive impairment using the SS-OIT. By analyzing the discriminative power of specific odors, we seek to improve the early detection and differentiation of cognitive decline. Additionally, understanding the influence of factors such as age, gender, verbal intelligence and depressive symptoms on olfactory performance may further enhance the diagnostic accuracy of olfactory assessments.

This study examines the relationship between olfactory impairment and cognitive decline across different diagnostic groups (HC, SCD, naMCI, aMCI, AD). It assesses whether differences exist in recognizing 16 odors and how cognitive impairment, along with factors such as age, gender and years of formal education (YFE) are associated to olfactory dysfunction.

The primary question is whether diagnostic groups differ in olfactory performance, using both total scores and individual items from the Sniffin’ Sticks test. A secondary objective is to evaluate the discriminative performance of individual Sniffin’ Sticks items considering influencing factors such as age, gender, verbal intelligence, depressive symptoms and cognitive status [13].

## Methods

### Study design and participants

This retrospective, single-center, cross-sectional study was based on clinical datasets collected between 2001 and 2022. Participants were included if they were at least 50 years old and had completed both neuropsychological and olfactory assessments within 90 days.

Exclusion criteria included any neurological disorder other than Alzheimer’s disease (AD), such as other forms of dementia (e.g., vascular dementia, Lewy body dementia), history of stroke, severe traumatic brain injury, brain tumors, hydrocephalus. Psychiatric illnesses were excluded. Mild-to-moderate comorbid depression was included in order to explore its independent contribution to olfactory performance, but its potential role as a confounding factor for cognitive impairment was statistically controlled. Nasal diseases such as sinusitis and coronavirus disease 2019 (COVID-19) infections in the anamnesis have been included.

Participants were grouped for primary regression analysis into AD versus non-AD (including SCD, naMCI, and aMCI), acknowledging that SCD and MCI may represent stages along the continuum toward AD but do not yet completely fulfil dementia criteria. The patient collective is represented by three diagnostic subgroups: SCD, MCI and AD, with participants allocated based on neurological and neuropsychological assessments as well as predefined criteria.

Individuals suffering from SCD were identified by applying the diagnostic criteria by Jessen et al., which state the necessity of subjectively perceived, persistent cognitive decline, despite neuropsychological test results, adapted to patients’ age, gender and YFE [9]. For diagnosing MCI, Petersen’s [34] criteria were considered, including subjective and objectively confirmed memory impairment, preserved general cognition, as well as daily functioning and the absence of dementia [34]. Patients with AD were diagnosed by adhering to the NINCDS-ADRDA (National Institute of Neurological and Communicative Disorders and Stroke and the Alzheimer’s Disease and Related Disorders Association) guidelines. Based on specific clinical and histopathological criteria AD can be classified into probable, possible, or definite Alzheimer’s dementia [35]. In 2011 the NINCDS-ADRDA guidelines were revised and expanded, by specifying the possible AD definition and integrating biomarkers to increase pathophysiological precision for possible and probable AD [36].

### Ethical aspects, benefit and risk evaluation

The patients participating in this study did not receive any direct benefits; however, the study contributes to advancing scientific understanding in the field. As this is a retrospective data evaluation, no associated risks were anticipated. The only identified risk, potential disclosure of sensitive data, was minimized through strict pseudonymization of patient protocols and robust access security measures. For the sole purpose of linguistic refinement and improving the organizational clarity of this report, the translation tool DeepL (Version 2025) [14] and the OpenAI language model ChatGPT (GPT‑4, [15]) were utilized. These tools were strictly limited to assisting with language-related and structural adjustments and were not employed in any capacity to process or analyze patient-sensitive data. This ensured full compliance with data protection regulations and ethical standards.

### Instruments

The Sniffin’ Sticks Odor Identification Test (SS-OIT) evaluates olfactory function through the identification of 16 common odors from 4 options [16]. Odor pens are held 1–2 cm in front of the nose for 3s and responses are given in a forced-choice format [17]. Scores range from 0–16, with ≥ 11 indicating intact olfaction and ≤ 10 reflecting hyposmia in individuals aged ≥ 50 years [18]. The SS-OIT was chosen for its strong psychometric properties (test-retest reliability > 0.80) and widespread clinical use across neurodegenerative populations. The SS-OIT takes about 8 min to perform.

The Mini-Mental State Examination (MMSE) is a standardized test for assessing cognitive functions, covering orientation, memory, attention, language, and visuospatial skills [19, 20]. Its brevity (5–10 min) makes it ideal for older patients and dementia screening [21].

The Vienna Visuoconstructional Test 3.0 (VVT3.0) evaluates visuoconstructive abilities using tasks like clock drawing, overlapping pentagons, and 3D cube replication. Scores range from 0–10, reflecting accuracy and quality [22–26]. The VVT3.0 can be obtained from www.psimistri.com.

The Wortschatztest (WST) measures verbal intelligence and premorbid cognitive abilities, aiding in dementia tracking. It consists of 42 recognition tasks, each with a target word and 5 distractors. Results are interpreted as IQ scores, with 85–115 representing normal performance [27, 28].

The Beck Depression Inventory-II (BDI-II) is a 21-item questionnaire assessing depressive symptoms based on DSM-IV criteria. Scores range from 0–63, with ≥ 14 suggesting clinically relevant depression. It is widely used in clinical and research settings due to its high reliability [29, 30].

The Neuropsychological Test Battery Vienna (NTBV-24) assesses cognitive performance in neurodegenerative diseases, covering attention, memory, executive function, language, and psychomotor speed across 24 subtests. Results include scores, times, and ratios, where higher scores indicate better performance, while longer times reflect impairment [31, 32]. The NTBV-24 has demonstrated robust construct validity and reliability in previous clinical samples. The NTBV-24 can be obtained from www.psimistri.com.

### Procedure

All assessments were conducted at the outpatient memory clinic of the Department of Neurology of the Medical University of Vienna. The neuropsychological battery (including MMSE, WST, NTBV-24) was usually completed first, followed by the SS-OIT and finally the BDI-II within the same or following session. The entire assessment procedure was completed within 90 days to ensure temporal validity of results. Test administrators were trained neuropsychologists.

### Statistical analysis

All statistical analyses were conducted using IBM SPSS® Statistics version 20.0 (IBM Corp., Armonk, NY, USA) and Microsoft Excel® for Windows® 11. The significance threshold was set at α = 0.05. Bonferroni correction was applied where appropriate to adjust for multiple comparisons (α* = α/k). Effect sizes were reported in accordance with Cohen’s classification (r ≥ 0.10 = small, ≥ 0.30 = medium, ≥ 0.50 = large). For Mann-Whitney U tests, effect size was calculated as r = z/√N.

Descriptive statistics included means (M), standard deviations (SD), medians (Mdn), interquartile ranges (IQR), minima and maxima for continuous variables. Frequencies and percentages were reported for categorical variables. Boxplots, profile plots, and bivariate scatterplots were used to visualize distributions and associations.

Group differences were examined using one-way Welch ANOVAs for normally distributed continuous data or Kruskal-Wallis tests for non-normally distributed data, χ^2^ tests were used for categorical variables, with degrees of freedom (df) and exact *p*-values reported.

Frequencies and percentages of correctly identified Sniffin’Sticks stimuli are reported. Differences are analyzed using Fisher’s exact test.

Diagnostic accuracy of the SS-OIT was evaluated using receiver operating characteristic (ROC) analysis. The area under the curve (AUC) was used to quantify discriminative performance, with higher values indicating better classification accuracy. The optimal cut-off was determined via the Youden Index (YI = sensitivity+ specificity −1). Positive predictive value (PPV), negative predictive value (NPV), and likelihood ratios (LR+ and LR−) were calculated to assess diagnostic utility. Specificity and sensitivity, the two main test quality criteria considering a dichotomous criterion, interact as antagonists; an increased specificity decreases sensitivity and vice versa. Furthermore, negative predictive value (NPV) and positive predictive value (PPV) are important screening parameters that describe the prediction of a criterion, whereas accuracy represents the concordance of detected negative and positive cases. Likelihood ratios (L + and LR−) were calculated and used as indicators for the practical applicability of a screening. If LR+ > 3 and LR− < 0.33, the assessment can be described as sufficiently accurate [33].

Binary logistic regression was performed to identify predictors of olfactory impairment, with model fit assessed using the Hosmer-Lemeshow test and explained variance evaluated via Nagelkerke’s R^2^. Wald statistics were used to test regression coefficients.

The regression model included five blocks in the following order: (1) SS-OIT total and selected odor scores, (2) age and gender, (3) verbal intelligence and YFE, (4) depression score (BDI-II) and (5) cognitive variables (NTBV factors). This stepwise order was based on theoretical and empirical considerations.

To explore the underlying factor structure of the NTBV-24, a principal component analysis (PCA) with orthogonal Varimax rotation was performed. The Kaiser-Meyer-Olkin (KMO) statistic was used to assess sampling adequacy (≥ 0.50 acceptable; ≥ 0.80 substantial). Components with eigenvalues ≥ 1 were retained. Factor scores were z‑standardized (M = 0, SD = 1) and interpreted based on communalities (h^2^ ≤ 1) and total explained variance (≥ 50%).

## Results

The sample comprised *N* = 737 participants, subdivided into healthy controls (HC; *n* = 134), subjective cognitive decline (SCD; *n* = 93), non-amnestic mild cognitive impairment (naMCI; *n* = 267), amnestic MCI (aMCI; *n* = 199) and Alzheimer’s disease (AD; *n* = 44). The overall gender distribution was 56.4% female, with no significant differences between diagnostic groups (χ^2^(4) = 9112, *p* = 0.058). Age did not significantly differ between men and women (z = −0.304, *p* = 0.761) but there were significant age differences across diagnostic groups (χ^2^(4) = 28914, *p* < 0.001), with AD patients being the oldest (Mdn = 75.1 years). The YFE did not differ significantly between groups (χ^2^(4) = 3217, *p* = 0.522). In terms of cognitive performance, verbal intelligence (WST-IQ) differed significantly across groups (F(4, 184.84) = 6793, *p* < 0.001), with the highest scores observed in the SCD group and the lowest in AD. Depressive symptoms (BDI-II) were significantly lower in the HC group compared to others (χ^2^(4) = 67,050, *p* < 0.001). Global cognitive function, assessed via the MMSE, showed a significant decline along the diagnostic continuum (χ^2^(4) = 159,962, *p* < 0.001), as did visuoconstructional ability, as measured by the VVT3.0 (χ^2^(4) = 29,322, *p* < 0.001). See Table 1 for detailed group comparisons.

**Table 1:** Demographic and clinical variables (cognitive performance, IQ, depressive symptoms and olfactory function). Healthy controls (HC), subjective cognitive decline (SCD), non-amnestic mild cognitive impairment (naMCI), amnestic MCI (aMCI), Alzheimer’s disease (AD), Mini Mental State Examination (MMSE), Vienna Visuoconstructive Test 3.0 (VVT3.0), Wortschatztest (WST-IQ), Beck Depression Inventory II (BDI-II)

	Demographic and clinical variables (cognitive performance, IQ, depressive symptoms and olfactory function)				
	HC (*n* = 134)	SCD (*n* = 93)	naMCI (*n* = 267)	aMCI (*n* = 199)	AD (*n* = 44)
*Age (years)*					
M ± SD	65.6 ± 10.6	67.1 ± 9.3	67.9 ± 9.3	68.9 ± 9.0	73.6 ± 7.4
min—max	50.1–93.6	50.3–90.4	50.0–89.8	50.4–90.2	50.0–83.5
Mdn (IQR)	64.1 (56.8; 73.0)	67.4 (60.7; 74.6)	69.2 (60.5; 74.7)	70.2 (61.6; 76.1)	75.1 (69.3; 78.7)
*Formal years of YFE (FYE)*					
M ± SD	12.2 ± 4.1	12.2 ± 4.1	12.2 ± 4.1	12.2 ± 4.1	12.2 ± 4.1
min–max	6–23	6–23	6–23	6–23	6–23
Mdn (IQR)	11 (9.0; 15.0)	11 (9.0; 15.0)	11 (9.0; 15.0)	11 (9.0; 15.0)	11 (9.0; 15.0)
*Gender*					
Male	47 (35.1%)	47 (35.1%)	47 (35.1%)	47 (35.1%)	47 (35.1%)
Female	87 (64.9%)	87 (64.9%)	87 (64.9%)	87 (64.9%)	87 (64.9%)
*MMSE (0–30)*					
*M* ± *SD*	28.68 ± 1.02	28.86 ± 1.15	28.08 ± 1.53	27.39 ± 1.70	24.66 ± 2.24
min–max	27–30	24–30	24–30	21–30	19–29
*Mdn* (IQR)	29 (28; 29)	29 (28; 30)	28 (27; 29)	27 (26; 29)	25 (24; 26)
*VVT3.0 (0–10)*					
*M* ± *SD*	9.87 ± 0.35	9.67 ± 0.77	9.40 ± 1.11	9.30 ± 0.95	8.05 ± 1.99
min–max	9–10	5–10	2–10	5–10	3–10
*Mdn* (IQR)	10 (10; 10)	10 (10; 10)	10 (9; 10)	10 (9; 10)	8 (7; 10)
*WST-IQ (µ* *=* *100,σ* *=* *15)*					
*M* ± *SD*	107.9 ± 11.5	113.8 ± 11.0	109.0 ± 12.6	107.1 ± 13.2	102.7 ± 16.6
min–max	77–133	84–139	74–139	77–139	60–129
*Mdn* (IQR)	107 (99; 118)	114 (106; 122)	110 (99; 118)	104 (99;118)	101 (92; 114)
*BDI-II (0–63)*					
*M* ± *SD*	5.22 ± 4.54	9.18 ± 6.94	11.46 ± 8.59	10.87 ± 8.97	11.12 ± 10.03
min–max	0–19	0–35	0–45	0–44	0–43
*Mdn* (IQR)	4 (2; 7)	7 (4.5; 13.5)	10 (5.5; 15)	9 (4; 15)	9 (4; 14)
*Sniffin’ Sticks (0–16)*					
M ± SD	12.81 ± 2.31	12.39 ± 2.50	11.99 ± 2.68	10.72 ± 2.87	8.50 ± 3.22
min–max	4–16	2–16	3–16	2–16	3–15
Mdn (IQR)	13 (12; 14)	13 (11; 14)	13 (11; 14)	11 (9; 13)	9 (6; 11)

*IQR* interquartile range, *Mdn* median

### NTBV factor analysis

To explore underlying cognitive dimensions, the 24 subtests of the Neuropsychological Test Battery Vienna (NTBV-24) were subjected to a principal component analysis (PCA) with orthogonal Varimax rotation and Kaiser normalization. The analysis included all 737 participants, and missing values (0.5%) were imputed using mean substitution. The Kaiser-Meyer-Olkin (KMO) coefficient of 0.867 confirmed the adequacy of the data for factor analysis and 6 independent factors were extracted, each with eigenvalues greater than 1, together explaining 73.8% of the total variance.

These dimensions were labeled based on the subtests with the highest loadings as follows: F1 language and verbal fluency, F2 attention, F3 verbal memory, F4 verbal attention, F5 vigilance, and F6 divergent reasoning. Factors F1 through F3 were scored in a positive direction, indicating that higher values represent better performance, whereas F4 through F6 were inversely scaled.

Subsequent comparison of these factor scores across diagnostic groups (HC, SCD, naMCI, aMCI, AD) using Welch-ANOVA revealed significant group differences on all six dimensions (*p* < 0.001 for F1–F3 and F5–F6; *p* = 0.001 for F4). In general, AD patients scored significantly worse than all other groups, especially on verbal memory (F3), attention (F2), and language fluency (F1), supporting the construct validity and clinical sensitivity of the NTBV-24 for detecting multidimensional cognitive impairment in neurodegenerative conditions. Extracted were six independent cognitive dimensions, all showing significant differences between diagnostic groups (*p* < 0.001). See Table 2 for details.

**Table 2:** Key values (M ± SD) of z‑standardized factor scores of the six NTBV dimensions considering HC and the diagnostic subgroups

Subgroup	F1 Language, verbal fluency (+)	F2 Attention (+)	F3 Verbal memory (+)	F4 Verbal attention (−)	F5 Vigilance (−)	F6 Divergent reasoning (−)
HC (*n* = 134)	0.160 ± 0.889	0.005 ± 0.889	0.396 ± 0.771	0.100 ± 0.995	−0.340 ± 0.546	−0.184 ± 0.867
SCD (*n* = 93)	0.460 ± 0.643	0.377 ± 0.748	0.397 ± 0.706	−0.241 ± 0.688	−0.174 ± 0.464	−0.241 ± 0.446
naMCI (*n* = 267)	−0.069 ± 0.894	−0.011 ± 1.006	0.435 ± 0.712	−0.045 ± 0.902	0.127 ± 1.042	0.055 ± 0.993
aMCI (*n* = 199)	−0.053 ± 1.136	−0.028 ± 1.045	−0.723 ± 0.890	−0.005 ± 1.142	0.080 ± 1.156	0.069 ± 1.154
AD (*n* = 44)	−0.801 ± 1.289	−0.619 ± 1.219	−1.414 ± 0.976	0.500 ± 1.258	0.273 ± 1.497	0.426 ± 1.294
*p*-value	< 0.001^**^	< 0.001^**^	< 0.001^**^	0.001^**^	< 0.001^**^	< 0.001^**^

^**^p ≤ 0.01

*HC* Healthy controls, *SCD* subjective cognitive decline, *naMCI* non-amnestic mild cognitive impairment, *aMCI* amnestic MCI, *AD* Alzheimer’s disease

### Olfactory function

Olfactory performance assessed by the Sniffin’ Sticks Identification Test (SS-OIT) significantly decreased with increasing cognitive impairment (χ^2^(4) = 98,446, *p* < 0.001, r = 0.37, medium to large effect). See Table 1 for detailed group comparisons.

Considering the SS-OIT score (range: 0–16; ≤ 10 = hyposmic), the proportion of normosmic and hyposmic individuals per subgroup was calculated. Across the total sample, 29.0% (95% confidence interval, CI 25.8–32.3%) exhibited hyposmia (see Table 3).

**Table 3:** Proportion of normosmic and hyposmic individuals per subgroup

Olfaction	Cognitive status, subgroup					Total (*N* = 737) (%)
	HC n = 134 (%)	SCD *n* = 93 (%)	naMCI *n* = 267 (%)	aMCI *n* = 199 (%)	AD *n* = 44 (%)	
Normosmic	113 (84.3)	75 (80.6)	205 (76.8)	116 (58.3)	14 (31.8)	523 (71.0)
Hyposmic	21 (15.7)	18 (19.4)	62 (23.2)	83 (41.7)	30 (68.2)	214 (29.0)
95% CI	9.5; 21.8	11.3; 27.4	18.2; 28.3	34.9; 48.6	54.4; 81.9	25.8; 32.3

*HC* Healthy controls, *SCD* subjective cognitive decline, *naMCI* non-amnestic mild cognitive impairment, *aMCI* amnestic MCI, *AD* Alzheimer’s disease

**Fig. 1:**
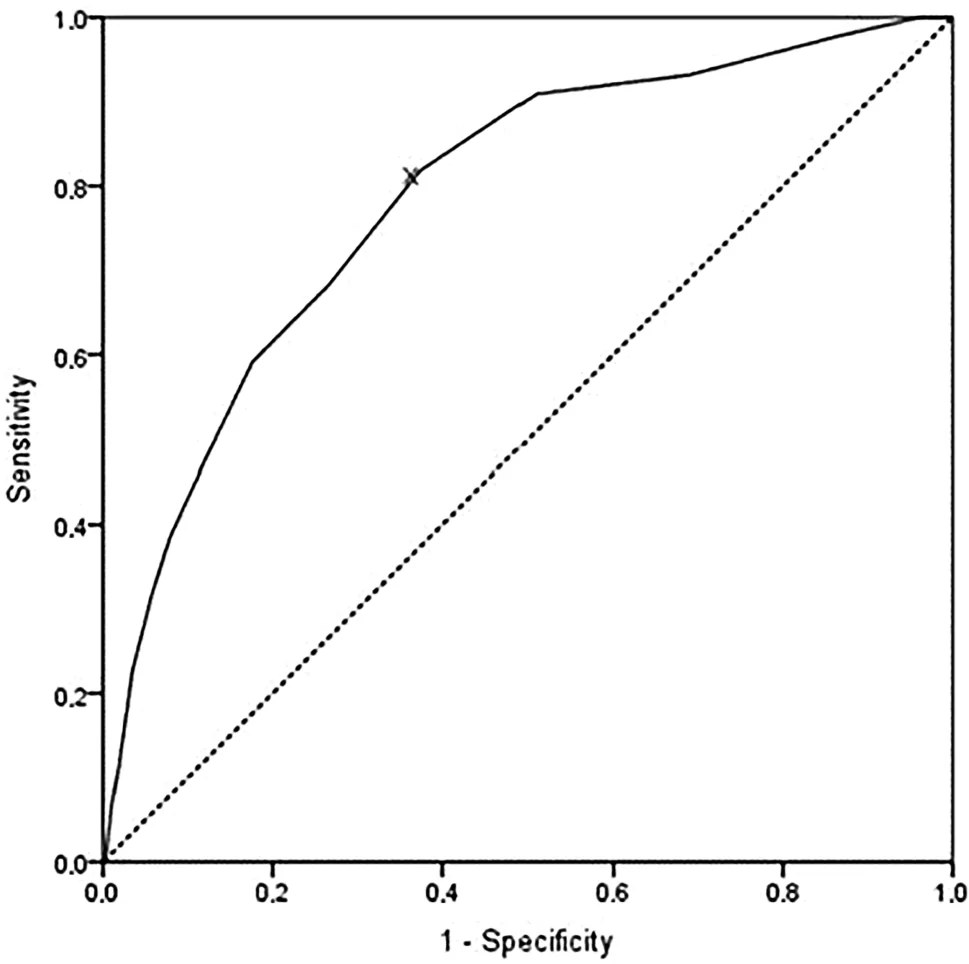
ROC-AUC: function of Sniffin’ Sticks scoring considering the criterion AD vs. non-AD (nNon-AD = 693, *N* = 737, nAD = 44)

To evaluate the diagnostic utility of the Sniffin’ Sticks Identification Test (SSIT) in predicting Alzheimer’s disease (AD), a receiver operating characteristic (ROC) analysis was conducted using the binary classification criterion non-AD versus AD. The analysis included all 737 participants (*n*_non-AD = 693, n_AD = 44) and revealed an area under the curve (AUC) of 0.788 (95% CI: 0.719–0.856), indicating good overall diagnostic accuracy. The optimal cut-off score for differentiating AD from non-AD cases was determined to be ≤ 11 points. This threshold yielded a sensitivity of 81.8% and a specificity of 62.6%, representing a balanced trade-off between identifying true positives and avoiding false positives. For settings where higher specificity is required, such as specialist referral or confirmation diagnostics, a stricter cut-off score of ≤ 8 was suggested, achieving 90% specificity at the cost of reduced sensitivity. The corresponding classification matrix demonstrated moderate diagnostic accuracy, with an overall accuracy of 63.8%. The positive predictive value (PPV) was 12.2%, whereas the negative predictive value (NPV) was notably high at 98.2%, indicating strong utility in ruling out AD in individuals with normal olfactory performance. The likelihood ratio for a positive test result (LR+) was 2.67, and for a negative result (LR–), 0.47. See Fig. 1 and Table 4 for details.

**Table 4:** Classification matrix: frequencies and proportions (%), indicating specificity, sensitivity, negative (NPV) and positive predictive value (PPV) of Sniffin’ Sticks scoring testing considering the non-AD versus AD group membership

		Predicted		Total (100%)
		“non-AD” (%)	“AD” (%)	
Observed	HC, SCD, naMCI, aMCI (non-AD)	434 = (62.6, Specificity)	259 (37.4)	693
		(98.2, NPV)		
	AD	8 (18.2)	36 = (81.8, Sensitivity)	44
			(12.2, PPV)	
	Total	442 (60.0)	295 (40.0)	737

Frequencies and percentages of correctly identified Sniffin’Sticks stimuli are reported (see Table 5). A detailed item analysis revealed that all single odors, except for cinnamon, turpentine and apple, showed strong discriminatory power between non-AD and AD participants, suggesting potential as early diagnostic indicators.

**Table 5:** Frequencies and percentages of correctly identified Sniffin’Sticks stimuli regarding two subgroups per Sniffin’ Sticks stimulus

Sniffin’ sticks (SN) item (correct answer)	Subgroup (*n*)		Total (737) (%)	Test-value χ^2^, *p*-value
	Non-AD (693) (%)	AD (44) (%)		
01 Orange	642 (92.6)	33 (75.0)	675 (91.6)	16,710, < 0.001^**^ (c.F.)
02 Leather	543 (78.4)	26 (59.1)	569 (77.2)	8724, < 0.003^**^
03 Cinnamon	441 (63.6)	22 (50.0)	463 (62.8)	3294, 0.070°
04 Peppermint	615 (88.7)	26 (59.1)	641 (87.0)	32,113, < 0.001^**^
05 Banana	610 (88.0)	29 (65.9)	639 (86.7)	17,549, < 0.001^**^
06 Lemon	394 (56.9)	17 (38.6)	411 (55.8)	5567, 0.018^*^
07 Liquorice	499 (72.0)	22 (50.0)	521 (70.7)	9670, 0.002^*^
08 Turpentine	348 (50.2)	18 (40.9)	366 (49.7)	1434, 0.231
09 Garlic	496 (71.6)	25 (56.8)	521 (70.7)	4347, 0.037^*^
10 Coffee	549 (79.2)	28 (63.6)	577 (78.3)	5912, 0.015^*^
11 Apple	282 (40.7)	13 (29.5)	295 (40.0)	2142, 0.143
12 Clove	523 (75.5)	24 (54.5)	547 (74.2)	9466, 0.002^**^
13 Pineapple	489 (70.6)	18 (40.9)	507 (68.8)	16,946, < 0.001^**^
14 Rose	601 (86.7)	26 (59.1)	627 (85.1)	24,881, < 0.001^**^
15 Aniseed	547 (78.9)	19 (43.2)	566 (76.8)	29,676, < 0.001^**^
16 Fish	623 (89.9)	28 (63.6)	651 (88.3)	27,685, < 0.001^**^

^**^p ≤ 0.01, ^*^p ≤ 0.05, ° *p* ≤ 0.10 (tendency); note: c.F. corrected by Fisher’s exact test

A multivariate binary logistic regression was conducted to evaluate whether olfactory identification performance (SS-OIT), alongside demographic and neuropsychological variables, could significantly predict AD diagnosis (criterion: AD = 1 vs. non-AD = 0). Predictors were entered hierarchically in five blocks (see Table 6).

**Table 6:** Multivariate binary logistic regression with olfactory identification performance, demographic and neuropsychological variables and Alzheimer’s disease (AD) diagnosis as criterion: AD = 1 vs. non-AD = 0. Predictors were entered in a hierarchical, blockwise fashion across five steps

Block	Predictor	*B*	*SE*	Wald χ^2^ (*df* = 1)	*p*-value	OR	95% CI	
							LB	UB
1	SN01 orange	0.358	0.557	0.414	0.520	1.431	0.481	4.261
	SN02 leather	0.281	0.410	0.467	0.494	1.324	0.592	2.960
	SN03 cinnamon	0.399	0.392	1.036	0.309	1.490	0.691	3.211
	SN04 peppermint	0.710	0.473	2.261	0.133	2.035	0.806	5.138
	SN05 banana	−0.035	0.494	0.005	0.944	0.966	0.367	2.542
	SN06 lemon	0.093	0.416	0.050	0.823	1.098	0.485	2.481
	SN07 liquorice	−0.174	0.433	0.161	0.688	0.840	0.360	1.963
	SN08 turpentine	0.177	0.393	0.203	0.652	1.194	0.552	2.581
	SN09 garlic	−0.246	0.422	0.341	0.559	0.782	0.342	1.788
	SN10 coffee	−0.327	0.460	0.506	0.477	0.721	0.293	1.777
	SN11 apple	−0.013	0.429	0.001	0.976	0.987	0.426	2.287
	SN12 clove	0.814	0.387	4.428	0.035*	2.256	1.057	4.812
	SN13 pineapple	0.651	0.407	2.568	0.109	1.918	0.865	4.255
	SN14 rose	1.057	0.445	5.655	0.017*	2.878	1.204	6.878
	SN15 aniseed	1.017	0.426	5.694	0.017*	2.765	1.199	6.377
	SN16 fish	−0.111	0.525	0.044	0.833	0.895	0.320	2.504
	Constant	−4.506	0.504	80.011	0.000	0.011	–	–
	Nagelkerke’s *R*^2^ = *18.7%*							
2	SN01 orange	0.312	0.557	0.314	0.575	1.366	0.459	4.067
	SN02 leather	0.284	0.414	0.472	0.492	1.329	0.591	2.989
	SN03 cinnamon	0.344	0.395	0.757	0.384	1.410	0.650	3.060
	SN04 peppermint	0.708	0.476	2.212	0.137	2.029	0.799	5.156
	SN05 banana	−0.038	0.490	0.006	0.939	0.963	0.369	2.514
	SN06 lemon	0.031	0.420	0.005	0.941	1.031	0.453	2.351
	SN07 liquorice	−0.178	0.434	0.169	0.681	0.837	0.358	1.958
	SN08 turpentine	0.146	0.396	0.136	0.713	1.157	0.533	2.512
	SN09 garlic	−0.277	0.424	0.426	0.514	0.758	0.330	1.741
	SN10 coffee	−0.376	0.462	0.663	0.416	0.686	0.277	1.699
	SN11 apple	−0.001	0.431	0.000	0.998	0.999	0.429	2.328
	SN12 clove	0.785	0.387	4.113	0.043*	2.192	1.027	4.681
	SN13 pineapple	0.578	0.408	2.008	0.157	1.782	0.801	3.964
	SN14 rose	1.009	0.449	5.036	0.025*	2.742	1.136	6.616
	SN15 aniseed	0.968	0.430	5.077	0.024*	2.634	1.134	6.115
	SN16 fish	−0.173	0.530	0.107	0.743	0.841	0.297	2.376
	Age (years)	0.030	0.023	1.633	0.201	1.030	0.984	1.078
	Gender	−0.129	0.388	0.110	0.740	0.879	0.411	1.882
	Constant	−6.329	1.588	15.891	0.000	0.002	–	–
	Nagelkerke’s *R*^2^ = *19.4%*							
3	SN01 orange	0.331	0.575	0.332	0.564	1.393	0.451	4.297
	SN02 leather	0.111	0.427	0.068	0.795	1.117	0.484	2.578
	SN03 cinnamon	0.295	0.402	0.539	0.463	1.344	0.611	2.957
	SN04 peppermint	0.716	0.480	2.225	0.136	2.046	0.799	5.241
	SN05 banana	−0.106	0.500	0.045	0.831	0.899	0.337	2.396
	SN06 lemon	0.080	0.424	0.036	0.850	1.084	0.472	2.490
	SN07 liquorice	−0.193	0.431	0.201	0.654	0.824	0.354	1.919
	SN08 turpentine	0.172	0.399	0.184	0.668	1.187	0.543	2.597
	SN09 garlic	−0.315	0.436	0.523	0.470	0.730	0.311	1.714
	SN10 coffee	−0.363	0.474	0.588	0.443	0.695	0.275	1.760
	SN11 apple	0.092	0.444	0.043	0.835	1.097	0.460	2.616
	SN12 clove	0.833	0.393	4.501	0.034*	2.300	1.065	4.964
	SN13 pineapple	0.613	0.414	2.190	0.139	1.846	0.820	4.156
	SN14 rose	0.953	0.456	4.369	0.037*	2.594	1.061	6.338
	SN15 aniseed	0.953	0.430	4.899	0.027*	2.593	1.115	6.027
	SN16 fish	−0.129	0.534	0.058	0.810	0.879	0.308	2.506
	Age (years)	0.033	0.023	1.957	0.162	1.033	0.987	1.082
	Gender	−0.258	0.399	0.418	0.518	0.772	0.353	1.689
	YFE	0.042	0.058	0.534	0.465	1.043	0.932	1.168
	WST-IQ	−0.040	0.016	5.926	0.015*	0.961	0.931	0.992
	Constant	−2.785	2.154	1.671	0.196	0.062	–	–
	Nagelkerke’s *R*^2^ = *22.1%*							
4	SN01 orange	0.328	0.570	0.332	0.565	1.389	0.454	4.247
	SN02 leather	0.097	0.428	0.051	0.821	1.102	0.477	2.547
	SN03 cinnamon	0.310	0.403	0.592	0.441	1.364	0.619	3.004
	SN04 peppermint	0.731	0.481	2.306	0.129	2.076	0.809	5.331
	SN05 banana	−0.082	0.499	0.027	0.870	0.921	0.347	2.449
	SN06 lemon	0.112	0.427	0.069	0.793	1.119	0.485	2.582
	SN07 liquorice	−0.209	0.433	0.232	0.630	0.812	0.347	1.896
	SN08 turpentine	0.151	0.402	0.141	0.707	1.163	0.529	2.555
	SN09 garlic	−0.335	0.439	0.581	0.446	0.716	0.303	1.692
	SN10 coffee	−0.383	0.476	0.648	0.421	0.682	0.268	1.734
	SN11 apple	0.084	0.446	0.035	0.851	1.087	0.454	2.604
	SN12 clove	0.823	0.393	4.390	0.036*	2.278	1.055	4.919
	SN13 pineapple	0.665	0.420	2.504	0.114	1.944	0.853	4.429
	SN14 rose	0.989	0.458	4.658	0.031*	2.688	1.095	6.597
	SN15 aniseed	0.935	0.430	4.722	0.030*	2.547	1.096	5.918
	SN16 fish	−0.148	0.538	0.076	0.783	0.862	0.300	2.475
	Age (years)	0.032	0.023	1.938	0.164	1.033	0.987	1.081
	Gender	−0.240	0.400	0.359	0.549	0.787	0.359	1.723
	YFE	0.044	0.058	0.582	0.446	1.045	0.933	1.170
	WST-IQ	−0.038	0.016	5.250	0.022*	0.963	0.932	0.995
	BDI-II	0.017	0.022	0.591	0.442	1.017	0.974	1.061
	Constant	−3.204	2.213	2.096	0.148	0.041	–	–
	Nagelkerke’s *R*^2^ = *22.3%*							
5	SN01 orange	0.049	0.746	0.004	0.948	1.050	0.243	4.535
	SN02 leather	−0.478	0.626	0.584	0.445	0.620	0.182	2.115
	SN03 cinnamon	0.882	0.547	2.603	0.107	2.416	0.827	7.056
	SN04 peppermint	1.214	0.678	3.212	0.073	3.368	0.893	12.707
	SN05 banana	−0.215	0.699	0.094	0.759	0.807	0.205	3.173
	SN06 lemon	−0.043	0.613	0.005	0.944	0.958	0.288	3.183
	SN07 liquorice	−1.036	0.660	2.466	0.116	0.355	0.097	1.293
	SN08 turpentine	0.499	0.547	0.833	0.362	1.648	0.564	4.818
	SN09 garlic	0.055	0.540	0.011	0.918	1.057	0.366	3.049
	SN10 coffee	−0.981	0.689	2.024	0.155	0.375	0.097	1.448
	SN11 apple	0.040	0.632	0.004	0.949	1.041	0.302	3.593
	SN12 clove	0.678	0.557	1.479	0.224	1.970	0.661	5.874
	SN13 pineapple	1.189	0.572	4.330	0.037*	3.285	1.072	10.070
	SN14 rose	0.847	0.640	1.754	0.185	2.333	0.666	8.172
	SN15 aniseed	0.073	0.554	0.017	0.895	1.076	0.363	3.190
	SN16 fish	−0.227	0.689	0.108	0.742	0.797	0.206	3.076
	Age (years)	−0.101	0.038	7.005	0.008**	0.904	0.838	0.974
	Gender	0.597	0.559	1.140	0.286	1.816	0.607	5.432
	YFE	−0.015	0.090	0.029	0.866	0.985	0.826	1.174
	WST-IQ	0.009	0.024	0.132	0.717	1.009	0.962	1.058
	BDI-II	0.007	0.028	0.053	0.818	1.007	0.952	1.064
	F1 language	−1.698	0.337	25.428	*<* 0.001**	0.183	0.095	0.354
	F2 attention	−1.236	0.309	16.044	*<* 0.001**	0.290	0.159	0.532
	F3 verb. memory	−2.295	0.376	37.271	*<* 0.001**	0.101	0.048	0.210
	F4 verb. attention	0.854	0.237	12.991	*<* 0.001**	2.348	1.476	3.736
	F5 vigilance	0.710	0.203	12.231	*<* 0.001**	2.033	1.366	3.026
	F6 div. reasoning	0.089	0.227	0.154	0.695	1.093	0.701	1.704
	Constant	−1.126	2.994	0.142	0.707	0.324	–	–
	Nagelkerke’s *R*^2^ = *59.9%*							

^**^p ≤ 0.01, ^*^p ≤ 0.05, °*p* ≤ 0.10

*WST-IQ* Wortschatztest, *BDI-II* Beck Depression Inventory II, *YFE* Years of formal education

Block 1: all 16 SS-OIT odor items were included. “Clove” (item 12), “Rose” (item 14), “Aniseed” (item 15), and “Pineapple” (item 13) emerged as strongest individual predictors (*p* < 0.001, odds ratios, OR, between 2.4 and 4.1). The model explained 18.7% of the variance in AD status (Nagelkerke’s R^2^ = 0.187).

Block 2: age and gender were added. These did not significantly improve model fit and did not attenuate the predictive power of the odor items.

Block 3: verbal intelligence (WST-IQ) and YFE was added. Verbal Intelligence had a protective effect (*p* < 0.01), with higher scores associated with a lower probability of AD diagnosis. The explained variance increased to 22.1%.

Block 4: depressive symptoms (BDI-II) were added. Although BDI-II scores were higher in clinical groups, they did not significantly predict AD status in the regression and did not alter existing associations.

Block 5: the six NTBV-24-derived cognitive factors were included. These markedly improved model fit, raising the explained variance to 59.9% (Nagelkerke’s R^2^ = 0.599). Of the six cognitive factors five (excluding psychomotor speed) contributed significantly. Importantly, the previously identified odors did not remain significant independent predictors; however, pineapple emerged as significant predictor demonstrating its robustness even after accounting for cognitive factors.

## Discussion

This study focused on odor-specific olfactory performance across different stages of cognitive impairment and its relationship with clinical covariates including age, gender, YFE, verbal intelligence, depressive symptoms and cognitive factors. The results provide novel insights into the role of individual odor items in the early detection of AD.

Among the 737 participants aged 50 years and older, olfactory performance, as measured by the Sniffin’ Sticks Identification Test (SS-OIT), declined significantly with increasing cognitive impairment. Based on a cut-off of ≤ 10 points, 68.2% of AD patients were classified as hyposmic supporting earlier studies and meta-analyses [37–42]. Depressive symptoms were significantly higher in cognitively impaired groups, while verbal IQ (WST-IQ) declined overall, with SCD patients outperforming even the healthy control group.

In ROC analysis based on the total SS-OIT score, the test achieved an AUC of 0.788, indicating good diagnostic accuracy. A cut-off of ≤ 11 points yielded a sensitivity of 81.8% and specificity of 62.6%, while a stricter threshold of ≤ 8 increased specificity to 90%, albeit at the cost of sensitivity. The overall classification accuracy was 63.8%, with a high negative predictive value (NPV = 98.2%), suggesting that normal olfactory performance can reliably rule out AD in this context. The positive predictive value (PPV = 12.2%) was remarkable low, indicating that olfactory dysfunction may not solely be associated with AD status. In the literature a LR+ > 3 and a LR− < 0.33 are discussed as showing that an assessment might be sufficiently accurate for screening purposes [33]. An LR+ of 2.67 and an LR− of 0.47 was found in the present study. These findings indicate a moderate effectiveness of the SS-OIT for the identification of AD further supporting the test’s potential as a valuable screening instrument in early detection and differential diagnosis of Alzheimer’s disease; however, for the screening of SCD and MCI stages the SS-OIT might not be effective enough and more sensitive (difficult) olfactory procedures are probably needed. Future studies should clarify this question.

Interestingly, the cut-off of ≤ 11 closely approximates the conventional hyposmia threshold (≤ 10) used in individuals aged 50 years and older [18]. This overlap raises the possibility that older individuals with borderline hyposmia could carry an increased risk of developing AD; however, the high NPV indicates that normosmic individuals in this age group are unlikely to exhibit underlying AD pathology, at least at the stage detectable by SS-OIT.

A central finding of this study lies in the item-specific analysis of odor identification. Patients with AD showed significantly lower correct identification rates for single odor items compared to non-AD individuals. Out of the 16 odors tested, clove, rose, aniseed after accounting for age, gender, education (FYE), verbal intelligence and depression, respectively, and pineapple after accounting for additionally cognitive factors, demonstrated the strongest discriminative power for identifying AD. These results remained significant in the logistic regression analyses, where these four odors independently contributed to AD classification. Their discriminative potential may reflect selective vulnerability of olfactory receptors or neural pathways affected early in the disease.

This corroborates previous findings, although different odors were emphasized across studies. For instance, one study reported peppermint, cinnamon, clove, banana, coffee, and rose as commonly affected [11] while another study found cinnamon and clove as sensitive markers for AD [6].

Such discrepancies could result from differences in cultural odor familiarity, test formats, or sample composition. Further research is needed to determine whether these odor-specific patterns are consistent across populations or reflect genetic dispositions or environmental influences.

The item-specific approach used in this study underlines the diagnostic relevance of individual odors, particularly those with strong predictive value for AD. These findings support the hypothesis that certain odorants may engage olfactory pathways or receptors more vulnerable to AD pathology. Implementing a reduced-item screening format focused on these key odors may enhance clinical feasibility without compromising diagnostic power. This approach could be particularly valuable in primary care or low-resource settings.

Although this study focused on differentiating AD from non-AD groups, future analyses should explore whether these odor-specific deficits are already detectable in earlier stages, such as SCD or aMCI. Identifying such patterns could support even earlier intervention. While our results point towards progressive olfactory decline across the cognitive spectrum, the discriminative power for early stages requires more targeted evaluation. Furthermore, a recently published genome-wide association meta-analysis (GWAMA) revealed gender-specific differences in the identification of specific odors: women exhibited an advantage in identifying orange and cinnamon, while men demonstrated an advantage in identifying pineapple [6].

Further research shows that olfactory decline in the general population is linked to AD-related blood biomarkers, supporting the hypothesis that common olfactory losses in ageing might partly reflect dementia-related processes [43].

Furthermore, the consistent impairment of specific odors in AD raises the question of whether such patterns reflect genetic or environmental influences, or both. For example, variability in olfactory receptor gene expression or cultural familiarity with certain smells might modulate odor perception and memory. This opens avenues for future research into gene-environment interactions and receptor-specific vulnerability as potential contributors to AD-related olfactory dysfunction.

### Limitations

Several limitations must be acknowledged. First, the sample size of the AD group was relatively small (*n* = 44), which may have affected statistical power in subgroup comparisons. Second, the cross-sectional design precludes conclusions about causal relationships or predictive validity. Third, although the SS-OIT is well-validated, it may not capture all dimensions of olfactory function (e.g., detection thresholds, discrimination). Cultural familiarity with specific odors was not assessed and may have influenced identification rates. Finally, despite statistical controls, comorbidities and medication effects could still act as unmeasured confounders, particularly in older clinical populations.

## Conclusion and outlook

This study highlights the diagnostic value of specific odors in the context of cognitive decline. Four odorants, clove, rose, aniseed, and pineapple, emerged as particularly effective in differentiating AD from non-AD individuals, even after adjusting for covariates. These findings underscore the potential of single-item analysis in enhancing olfactory screening tools.

Given the strong link between odor misidentification and AD, the development of reduced-item, culturally validated olfactory screening tools is a promising direction. Future studies should investigate genetic and environmental correlates of selective odor impairment, examine cross-cultural applicability, and explore whether these patterns hold in preclinical and at-risk populations.

## Data Availability

The datasets generated during and analyzed during the current study are available from the corresponding author on reasonable request.
